# Carbon storage estimation in a secondary tropical forest at CIEE Sustainability Center, Monteverde, Costa Rica

**DOI:** 10.1038/s41598-021-03004-5

**Published:** 2021-12-06

**Authors:** Alexandra Paniagua-Ramirez, Oliwia Krupinska, Vicki Jagdeo, William J. Cooper

**Affiliations:** 1 Council On International Educational Exchange, Sustainability Center, Monteverde, Costa Rica; 2 Lehigh University, Bethlehem, Pennsylvania USA; 3 Pierella Rainforest Reclamation Project, 324 Main Street, Suite 1322, Laurel, MD 20707 USA

**Keywords:** Environmental sciences, Environmental impact, Plant sciences, Natural variation in plants

## Abstract

Secondary growth tropical rainforests have the potential to sequester large amounts of atmospheric carbon dioxide and as such are an important carbon sink. To evaluate a local forest, a Carbon Neutrality Program was initiated at the Council on International Educational Exchange, San Luis Campus, Monteverde, Costa Rica. The study was conducted on 50 hectares of forest classified as Premontane Wet Forest. The forest, part of the Arenal-Monteverde Protected Zone, is estimated to be aproximately 50 years old and is in the upper regions of the San Luis valley at 1100 m elevation. Assessment of the carbon stock in trees was carried out in two permanent, 1 hectare plots, 100 m by 100 m, Camino Real and Zapote. The plots were divided into 25 subplots, 20 m by 20 m totaling 400 m^2^ per subplot. Ten subplots in each area were studied which represented 1.6% the total surface area of the forest. All of the trees were measured within the subplots that had a diameter at breast height ≥ 10 cm and the height of 10% of the trees measured. The estimated total CO_2_ sequestered by the campus forest was 18,210 ton (in 2019).

## Introduction

Carbon dioxide is recognized as the primary greenhouse gas of the four gases that contribute the most to global warming. They include, carbon dioxide, CO_2_ (81%); methane, CH_4_ (10%); nitrous oxide, N_2_O (7%); and, halogen containing gases, CFCs (chlorofluorocarbons) (3%)^1–3^. Carbon dioxide results primarily from the combustion of fossil fuels which are derived from plants. Coal originates from decayed plant material occurring in swampy environments and is buried over geologic time. Oil originates from the phytoplankton and other organic matter that accumulates in oceanic sediments and after burial migrates underground to reservoirs. Coming full circle, Dyson in 1977 was the first to propose removing CO_2_ from the atmosphere using plant photosynthesis^4^. The present study further explores the importance of carbon capture and sequestration in a neotropical rainforest.

The concentration of carbon dioxide in the atmosphere has increased from preindustrial concentrations of approximately 280 ppm (parts per million by volume)^5^ to the present 419 ppm worldwide^3,6–8^. As atmospheric CO_2_ has become a central focus in climate change science the study of all aspects of the biogeochemical carbon cycle is the focus of much research^1^. One of the most important aspects of the carbon cyle, is the estimation of the halflife of CO_2_ in the atmosphere^9–13^. However, because of the complexity of the carbon cycle, the estimates range from 30 to 95 years^14^. If one considers the long CO_2_ concentration tail in some models, it has been suggested that the halflife could be as long as 300 years^15^. Better constraining these estimates is a very active area of research and it is possible that the Climate Model Intercomparison Project now in its sixth iteration (CMIP6) will lead to better estimates of atmospheric CO_2_ lifetimes^16,17^.

Limiting climate change will require that the atomspheric concentration of CO_2_ is stabilized^18,19^. The IPCC (2018) report entitled “Global Warming of 1.5 °C” emphasizes that new or renewed efforts are needed to reach the goal of stabilizing atmospheric concentrations of CO_2_^20^. Studies have been published addressing stabilizing atmospheric CO_2_ concentrations^21–24^. However, the concentration of CO_2_ continues to rise a little over 2 ppm per year and forests are likely to play an important role in the future^5^.

The importance of forests was reviewed in a report prepared by the World Resources Institute^25^, and more recently by the IPCC^26,27^. Forests (or planting forests) can be thought of as reverse engineering human deforestation and providing a natural sink for CO_2_^28–30^. It is estimated world-wide, that of the 4.06 billion hectares of forest, 45% (1.8 billion hectares) are tropical forests^31,32^. Tropical forests provide the highest potential for carbon capture and sequestration because they have the highest carbon density of all forests^33^.

Many institutions are studying their environmental impact by initiating carbon neutral programs. To reduce their carbon emissions and offset those emissions which they can not reduce, a potential first step is the preservation of forests on-premises. In this approach, growing trees sequesters carbon. The objective of this study was to calculate the carbon stored by trees in 50 hectares of forests by studying in detail two one-hectare plots at the Council on International Educational Exchange (CIEE) San Luis campus, Monteverde, Costa Rica.

## Results

**Figure 1:**
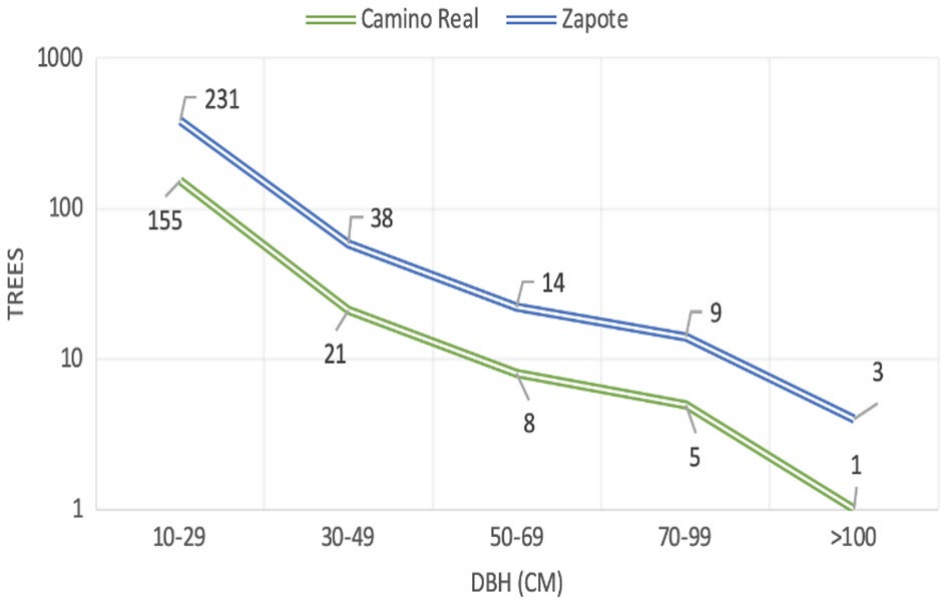
The distribution of trees based on diameter at breast height (DBH) for the Camino Real and Zapote plots.

The focus of this study was the above ground carbon captured. This was designed to define the carbon capture and sequestration of a secondary forest at one point in time. Our data, summarized in Fig. 1, shows that both plots have predominantly young tree growth with the diameter at breast height (DBH) between 10 and 30 cm and the distribution of tree sizes is approximatley the same. This confirms their young age and secondary forest status. However, Zapote (the top line) appears to be somewhat older and had a higher number of trees, when compared to Camino Real, at every DBH.

A frequency distribution suggested that the four data points of DBH > 100 cm did not fit the data and suggested that these four trees were older that the rest of the plots and were considered outliers. Therefore, they were not included in the estimation of basal area for either of the plots.

To find the carbon storage within the plots, it was necessary to calculate the basal area of the subplots, which referred to the total surface area covered by tree trunks in relation to the surface area of a plot. A statistical analysis for “tree basal area” was performed to give the average basal areas per plot. The standard deviation, standard error, coefficient of variation, and, sampling mean error in basal areas were no more than 20%, at a confidence interval of 95% using Student´s Distribution Table (n − 1). After calculating the carbon stored in each subplot the value for each hectare was determined and then the result was multiplied by the number of hectares of each forest.

**Figure 2:**
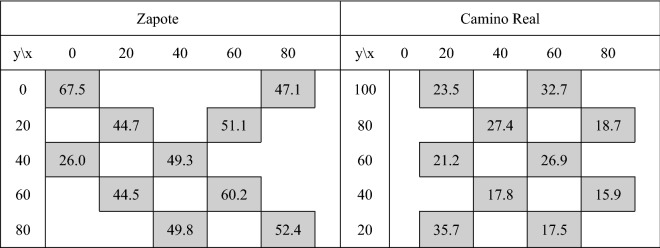
Basal areas (m^2^ ha^−1^) for the Zapote and Camino Real plots showing subplot locations.

The basal area results for Zapote and Camino Real plots are summarized in Fig. 2, for each subplot map the basal average is given. It is noted that the Zapote (average area 49.3 m^2^ ha^−1^) plot had a much greater tree cover than Camino Real (average area 23.7 m^2^ ha^−1^). The results of the basal areas of each plot are consistent with the plot assignments given based on the “Google map” tree densities.

In total there were 60 diferent species of trees in both plots. In the Zapote plots the most common species were *Eugenia guatemalensis* (21), *Dendropanax arboreus* (18), *Cecropia obtusifolia* (18), *Inga punctata* (28), and *Nectandra salicina* (38). In Camino Real, there was a substantial number of *Inga punctata,* in addition to the four above.

As seen in Table 1, based on the National Forest Inventory^34^, a secondary forest has approximately 204 tonnes ha^−1^ of CO_2_ while a mature forest has 447 tonnes ha^−1^. The Camino Real plot with 243 tonnes ha^−1^ was close to that of a secondary forest while the Zapote plot with 518 tonnes ha^−1^ was closer to the mature forest of 447 tonnes ha^−1^. Zapote’s high basal area and carbon storage indicates that it is an older secondary forest with many young trees competing with the larger trees.

**Table 1:** Calculated values for the two permanent plots and the average values reported for a secondary forest and mature forest.

Variable	Zapote plot	Camino real plot	Typical secondary forest (National Forest Inventory of Costa Rica)^34^	Typical mature forest (National Forest Inventory of Costa Rica)^34^
Numbers of trees (ha^−1^)	737	475	415	469
Average basal area (m^2^ ha^−1^)	49.3	23.7	19.3	32.3
Volume (m^3^ ha^−1^)	560	269	159	338
CO_2_ (ton ha^−1^)	518	243	204	447

Table 2 summarizes the data and shows that the Zapote plot collected slightly over twice the CO_2_ of the Camino Real plot. This is reasonable because of the differences in the basal areas of the two plots. These two plots are much different from each other which suggests that they were good representatives of the total 50 hectare campus forest area. Taking the average CO_2_ per hectare of each plot and extrapolating to the campus forests provides an estimate of the total carbon sequestration.The estimation of the total CO_2_ collected by the campus forest to date is aproximately 18,210 tonnes.

**Table 2:** Estimation of the carbon storage in the CIEE forest, Monteverde, Costa Rica.

Plot	Average ton of CO_2_ plot^−1^	Average ton of CO_2_ ha^−1^	Corresponding hectares
Zapote	11,400	518	22
Camino real	6810	243	28
Total tonnes CO_2_ in the forest	18,210		

### Limitations

The tags of some trees (< 20 individuals) had fallen off; therefore, they were identified based on past measurements and locations recorded, if they could not be identified, they were marked as new trees. This should not affect the final results of our analysis since we are looking at the total basal area rather than individual trees.

In the Zapote plot, there were no marks on trees which would indicate previous measurements, so the trees were measured according to the Protocol, DBH at 1.3 m from the base unless a tree presented irregularities that required a different measurement location. However, 2018 measurements were taken 10 cm below tags. This likely led to a systematic underestimation of the forest growth, since many trees had tags which were located at 1.3 m.

The accuracy of height measurements was restricted by the tool and our ability to see the top of a tree canopy and the length of the measurement stick. Trees were generally chosen at random, but heights of trees above 40 ft (12.1 m) had to be estimated by eye and were subject to greater error. This could potentially lead to an underestimation of average tree height.

Other problems in assessing different factors; for example, form factor or basic specific weight of each tree, derived from the lack of data on non-commercial trees led to higher uncertainty.

## Discussion

The numbers calculated for total carbon stored are typical for secondary forests. The Camino Real area historically has been more interrupted by human activity than the Zapote plot which is why it has less trees, less basal area and ultimately less carbon volume. The Zapote plot, on the other hand, has been less interrupted, so the trees in this permanent plot have had more time to grow and the tree density is lager at each DBH. This is additionally supported by the Google Earth images of both plots, where one can clearly see that the sections labeled as Zapote are denser while Camino Real is more scarce and divided by pastures. There are approximately 518 tonnes ha^−1^ of CO_2_ stored in the Zapote plot and 243 tonnes ha^−1^ of CO_2_ in Camino Real plot (Table 1).

Secondary forests, like the permanent plots of Zapote and Camino Real, have great potential to sequester carbon through tree growth, as they gain biomass more rapidly than older primary forests. Now that there is a baseline for the volume of carbon stored in these permanent plots, we can calculate the carbon sequestered in subsequent years. This can be done through the same measurement procedure as described in this study, comparing the initial and final carbon storage. This work could be used in the future calculations of carbon sequestration for CIEE’s carbon neutrality program and as a reference for other institutions which also wish to understand carbon sequestration of secondary forests, potentially for their own carbon neutrality calculations.

In the future the sample size should be increased. This study represented 1.6% of the total forested area at the lower end of 1–5% of the total area recommneded for studies of this nature^50^**.**

## Methods

### Study area

This study was conducted on the CIEE San Luis campus, in the monitoring plots which were first established in 1997 and 2014 by Diana and Milton Lieberman on the grounds of Ecolodge San Luis and Biological Station (University of Georgia, Costa Rica Campus) and purchased by CIEE in 2019. The site has 50 hectares of forest in the upper regions of the San Luis valley at 1100 m above sea level^35^. It is classified as Premontane Wet Forest according to Holdridge and is part of the Arenal-Monteverde Protected Zone^36,37^. The property borders the Monteverde Cloud Forest Reserve and the Children’s Eternal Rainforest and hosts a unique assemblage of flora and fauna from the cloud forest above and dry forest below. In Costa Rica, the Premontane Wet Forest life zone faced rapid deforestation in the mid to late 1900s as vast areas were converted to pasture. As a result, what remains of the forests is second only to the tropical dry forest as the most heavily altered and reduced life zone in the country^38^.

**Figure 3:**
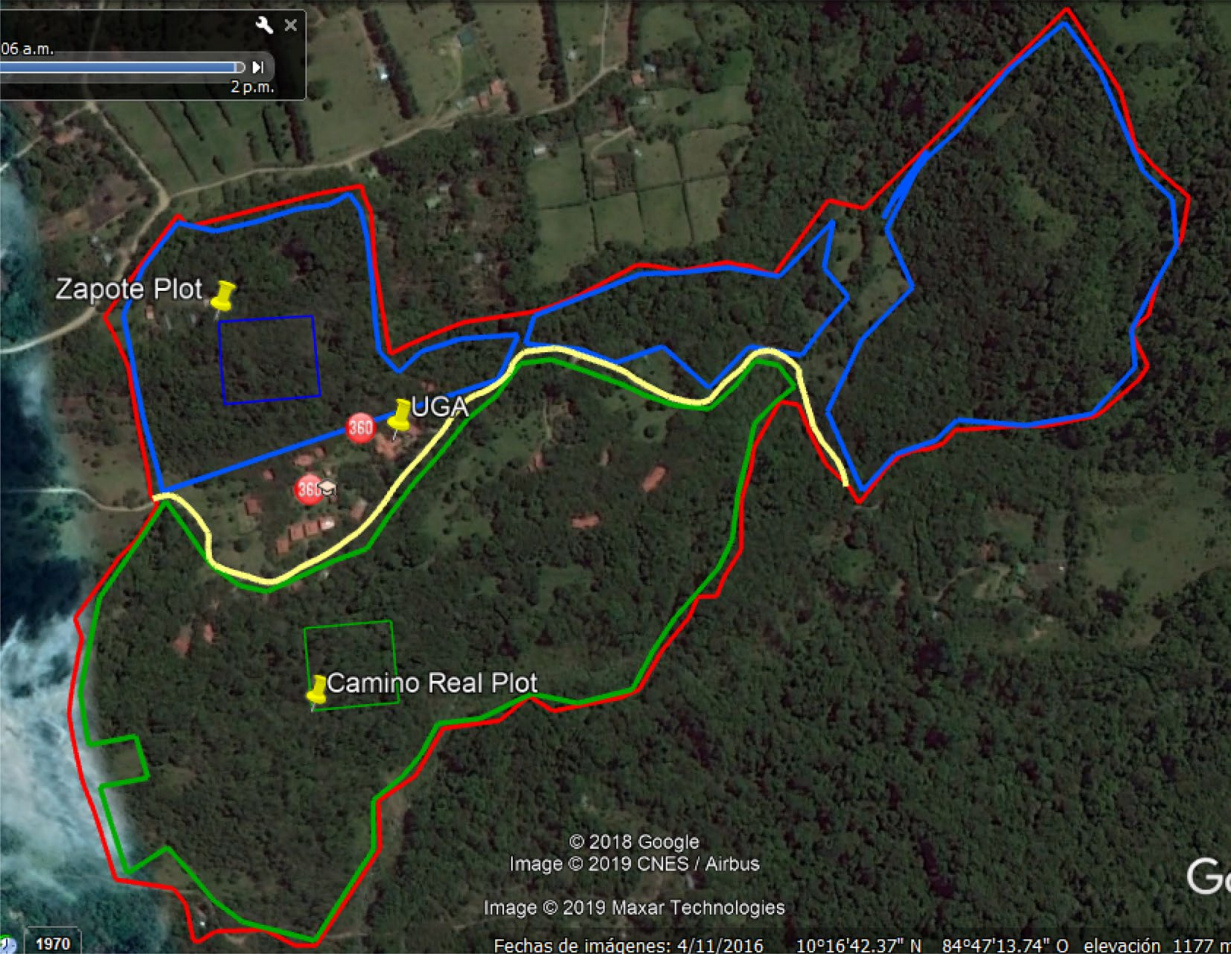
Map of the campus and location of Zapote and Camino Real Plots. Map of the campus outlined in red, the forest areas corresponding to Zapote in blue and Camino Real in green. (2018 Google, Image ©2019 Maxar Technologies).

The study area is based on two pre-established plots on the campus premises, Camino Real and Zapote. Both plots are 100 m × 100 m (1 ha) divided into 25 subplots each. Camino Real is a plot located alongside the Camino Real trail with its southwest point located at 10° 16.800′ N, 84° 47.952′ W. It was established on a section of secondary forest south of main campus with DBH measurements beginning in 2014. The Zapote plot is on the north side of campus, by the Zapote trail, and has its northwest corner located 10° 17.321′ N, 84° 47.800′ W. Historically, much of the property was used for coffee, guava, banana, and pasture land until the 1970s. However, the area within the Zapote plot was less intensively used and more closely resembles primary forest when compared to the Camino Real plot on the lower slopes of the property near Rio Alondra as shown in^39^ Fig. 3.

### Methods

Assessments of biomass, carbon capture and sequestration, is an active area of study and several methods have been published^30,39–49^. In this study the estimation of carbon stock was derived directly by dasometric variables of trees through measuring sample tree attributes in the field, such as diameters (DBH), tree heights and using an allometric equation. All the measurements in this study were conducted in accordance with the Costa Rican National Standard INTE/DN 03:2016 Methodology for Quantification and Reporting Greenhouse Gas Removals Resulting from Forestry Activities (National Standard INTE/DN 03:(2016)^50^. This is the official standard for developing inventories for the Carbon Neutrality Program, and the Protocol for the Establishment and Measurement of Permanent Sampling Plots in Natural Forest^39^.

### Experimental design

**Figure 4:**
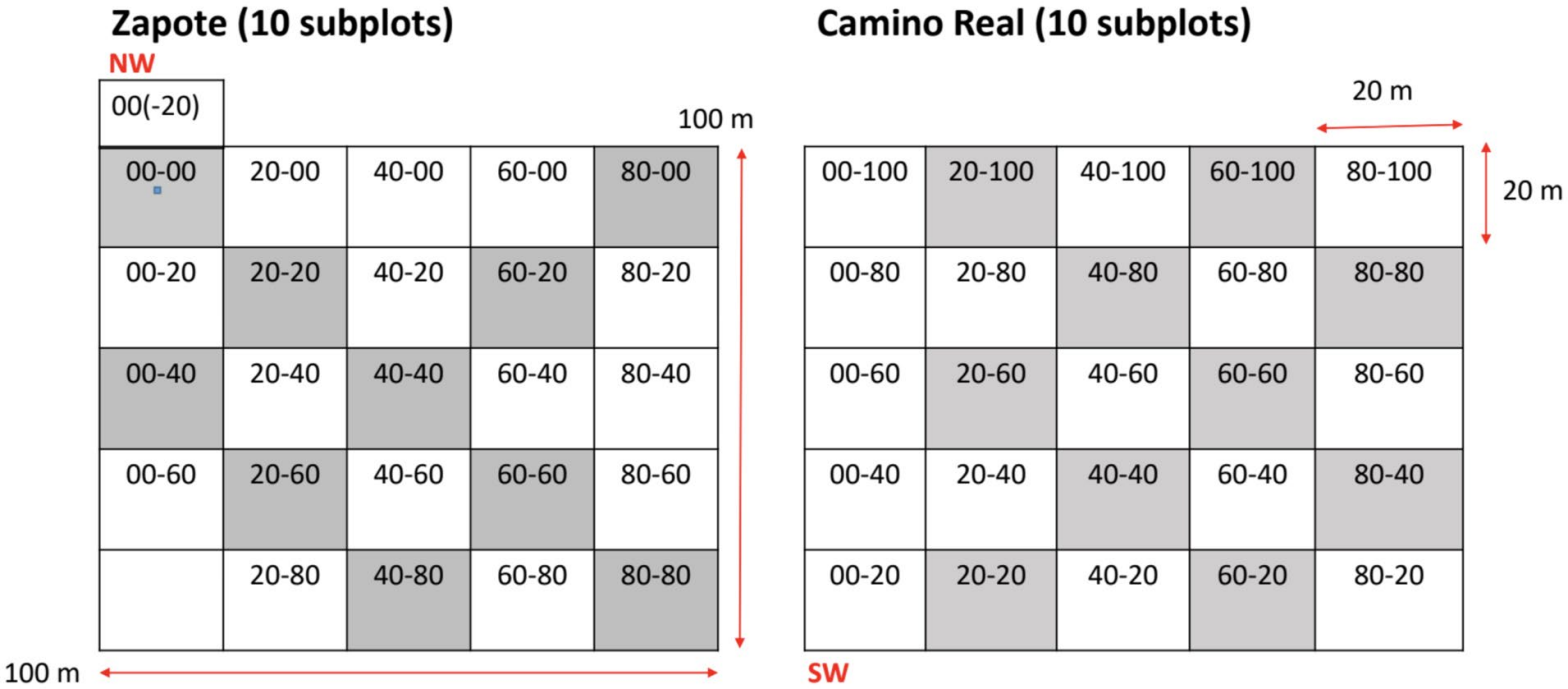
Diagramatic representation of the subplots chosen for this study are the shaded subplots.

Each one-hectare plot was divided into 25 subplots and then 10 subplots, 20 m by 20 m designated for sampling (Fig. 4). This design represented 40% of the two one-hectare plots studied and 1.6% of the total forest area (20 plots each 0.04 ha/50 ha^−1^ = 1.6%).

### Plot reconnaissance

The plots, Zapote and Camino Real, were first set up in 2014. Therefore, prior to going to the field it was necessry to examine previous data collected. This included a list of the trees with DBH ≥ 10 cm, their assigned number, species, previous recorded diameter measurements and tree conditions as well as their coordinates. During the reconnaissance if a tree needed a new tag; for example, if the tag was missing, broken, fallen, or covered by tree growth, a new tag was made and afixed to the tree for future reference.

The northwest corner of the Zapote plot was identified with GPS, then a compass was used to scout subsequent subplots and checked with the permanent plot map. Where possible the visible markers at the corners of the subplots were located. In each subplot, tree tag numbers and referenced the data sheet were used to double check that the tree was in the assigned subplot. Since most tree measurement locations were not marked the tree was numbered, DBH measured (1.3 m from the base of the tree) and marked with paint for future reference.

The southwest corner of the Camino Real plot was identified with GPS and then a compass was used to scout subsequent subplots. In addition, the tree tags in Camino Real indicated the given subplot number (1–25) on the tag which helped to find the correct location of the plot as well. The tree measurement locations in Camino Real were well marked with paint and the DBH was verified.

### Tree measurement procedure

The measurements for this study were conducted during the months of June to August 2019. All of the trees were measured that had a DBH ≥ 10 cm and the height of at least 10% of the trees in the plot.

Tree height was determined using a stick measure (telescopic cylinder for heights) to get an overall average tree height in the given subplot. If a tree was over the top of the pole (40 feet, 12.1 m) either the height was estimated or the average height within the subplot was used. For each tree, its condition was recorded according to the conditions specified in Sánchez-Monge^39^.

If a tree looked like a new recruit, its DBH was measured to see if it was actually ≥ 10 cm. A new recruit was marked with colored tape with a new tree number, which was assigned as the next number in the subplot based on the datasheet. Then the tree number, diameter, condition, and location based on its proximity to other trees was recorded.

If a tree could not be found based on the coordinates on the datasheet the condition code of 16 (tree not found) would be written down. If a previously measured tree was dead, it would be recorded as dead and not measured.

### Calculation of CO_2_

The following allometric equation, Eq. (1), was used to calculate the above ground carbon stored within the plots:

1 $${\text{CO}}_{2} {\text{e}} = {\text{DBH}}^{2} *(\pi {/}4)*h*{\text{FF}}*{\text{CF}}*{\text{BSW}}*{\text{BEFa}}*{\text{BEFg}}*3.67$$

To estimate the amount of carbon captured, CO_2_e, a conversion factor of one ton of forest biomass has approximately 0.5 tonnes of carbon, was used.

The basal area is DBH^2^ * (π/4), and *h* is the tree height.

However, a tree is not a perfect cylinder so multiple factors have to be applied according to IPCC Guidelines the “fitting factors” are^51^:

- FF is the “form fitting” factor which takes into consideration the change in diameter in relation to height. The FF varies according to the tree species but, if the species could not be identified, the IPCC allows a FF of 0.7 in forest trees, meaning the average diameter is 70% of the DBH.
- CF is the carbon fraction which represents the estimated volume of carbon stored in the trunk and is considered to be approxcimately 50%, or 0.5.
- BSW is the basic specific weight which refers to the weight of the dry wood in a tree. It varies by species and is related to the density of the wood. If the species of the tree is unknown, the IPCC recommends using 0.5^51^.
- BEFa and BEFg refer to the volume of the branches and leaves (BEFa) and roots (BEFg). For above-ground biomass the estimate for both was 1.2 of the volume of the trunk, calculated by the basal area times height.

Finally, 3.67 is the ratio of the weight of carbon in carbon dioxide. This means that one tonne of carbon is equivalent to 3.67 tonnes of CO_2_ (obtained based on the molecular weights of CO_2_ and carbon, of 44/12).

### Extrapolating to the 50 hectares

To estimate the total amount of carbon captured, a Google Earth image of CIEE Campus was used to estimate how much of the forest was similar to the Zapote, a mature plot, and how much was like Camino Real, an apparently more disturbed plot. Any dense tree areas were defined as more mature, while the areas with more pastureland and scarce growth were defined as younger, corresponding to Zapote and Camino Real, respectively. Figure 3 shows a map of the campus along with mature areas in blue and less mature areas in green. It was concluded that the landscape similar to Zapote, was 22 ha and that similar to Camino Real was 28 ha.

## Data Availability

All of the supporting data for this paper are available in the original research notebooks and data files at CIEE.

## References

[1] Houghton RA (2007). Balancing the global carbon budget. Annu. Rev. Earth Planet. Sci..

[2] US Environmental Protection Agency, Greenhouse Gas Emissions. Inventory of U.S. Greenhouse Gas Emissions and Sinks (2019). https://www.epa.gov/ghgemissions/overview-greenhouse-gases. Accessed Feb 2021.

[3] Buis, A. The atmosphere: Getting a handle on carbon dioxide. Sizing up humanity’s impacts on Earth’s changing atmosphere: a five-part series. Part Two. NASA Global Climate Change (2019). https://climate.nasa.gov/news/2915/the-atmosphere-getting-a-handle-on-carbon-dioxide/. Last updated 9 Feb 2021.

[4] Dyson FJ (1977). Can we control the carbon dioxide in the atmosphere?. Energy.

[5] Lindsey, R. Climate Change: Atmospheric Carbon Dioxide. https://www.climate.gov/print/8431 (2020).

[6] Blunden, J. & Arndt, D. S., Eds. State of the Climate in 2018. *Bull. Am. Meteorol. Soc.*, **10**(9), Si-S305. 10.1175/2019BAMSStateoftheClimate.1 (2019).

[7] Bruhwiler L (2021). Observations of greenhouse gases as climate indicators. Clim. Change.

[8] Butler, J. H. & Montzka, S. A. The NOAA Annual Greenhouse Gas Index (AGGI). Global Monitoring Laboratory, Earth System Research Laboratories, R/GMD, 325 Boulder CO 80305-3328, 13 pp. (2020).

[9] Lashof DA, Ahuja DR (1990). Relative contributions of greenhouse gas emissions to global warming. Nature.

[10] Moore B, Braswell BH (1994). The lifetime of excess atmospheric carbon dioxide. Global Biogeochem. Cycles.

[11] Jacobson MZ (2002). Control of fossil-fuel particulate black carbon and organic matter, possibly the most effective method of slowing global warming. J. Geophys. Res..

[12] Jacobson MZ (2005). Correction to “Control of fossil-fuel particulate black carbon and organic matter, possibly the most effective method of slowing global warming”. J. Geophys. Res..

[13] Archer D (2009). Atmospheric lifetime of fossil fuel carbon dioxide. Annu. Rev. Earth Planet. Sci..

[14] Matthews HD, Caldeira K (2008). Stabilizing climate requires near-zero emissions. Geophys. Res. Lett..

[15] Archer D (2005). Fate of fossil fuel CO_2_ in geologic time. J. Geophys. Res..

[16] Eyring V (2016). Overview of the coupled model intercomparison project phase 6 (CMIP6) experimental design and organization. Geosci. Model Dev..

[17] Simpkins G (2017). Progress in climate modelling, interview with Veronika Eyring. Nat. Clim. Change.

[18] Houghton RA (2013). The emissions of carbon from deforestation and degradation in the tropics: past trends and future potential. Carbon Manag..

[19] Houghton RA, Byers B, Nassikas AA (2015). A role for tropical forests in stabilizing atmospheric CO_2_. Nat. Clim. Chang..

[20] IPCC Global Warming of 1.5 °C. An IPCC Special Report on the impacts of global warming of 1.5 °C above pre-industrial levels and related global greenhouse gas emission pathways, in the context of strengthening the global response to the threat of climate change, sustainable development, and efforts to eradicate poverty (eds Masson-Delmotte, V. *et al.*) (2018).

[21] Azar C, Rodhe H (1997). Targets for stabilization of atmospheric CO_2_. Science.

[22] Azar C, Lindgren K, Larson E, Möllersten K (2006). Carbon capture and storage from fossil fuels and biomass—costs and potential role in stabilizing the atmosphere. Clim. Change.

[23] Hansen J (2008). Target atmospheric CO_2_: Where should humanity aim?. Open Atmos. Sci. J..

[24] Kriegler E (2018). Pathways limiting warming to 1.5°C: a tale of turning around in no time?. Philos. Trans. R. Soc. A.

[25] Faeth, P., Cort, C. & Livermash, R. Evaluating the carbon sequestration benefits of forestry projects in developing countries. World Resources Institute, Washington DC. 96 pp (1994).

[26] IPCC Third Assessment Report Climate Change 2001: Mitigation. A Report of Working Group III of the Intergovernmental Panel on Climate Change. Chapter 4. Technological and Economic Potential of Options to Enhance, Maintain, and Manage Biological Carbon Reservoirs and Geo-Engineering. Kauppi, P. & Sedjo, R. (Lead Authors), 301–343 (2001).

[27] IPCC *Climate Change 2007: Mitigation. Contribution of Working Group III to the Fourth Assessment Report of the Inter- governmental Panel on Climate Change* (eds Metz, B., Davidson, O. R., Bosch, P. R., Dave, R. & Meyer, L. A.) (Cambridge University Press, Cambridge, United Kingdom and New York, NY, USA, 2007).

[28] Houghton RA (1983). Changes in the carbon content of terrestrial biota and soils between 1860 and 1980: a net release of CO_2_ to the atmosphere. Ecol. Monogr..

[29] Evans, J. & Turnbull, J. W. *Plantation Forestry in the Tropics: The Role, Silviculture, and Use of Planted Forests for Industrial, Social, Environmental, and Agroforestry Purposes* 3rd edn, 488 pp (Oxford University Press, 2004).

[30] Chou SW, Gutiérrez-Espeleta E (2013). Ecuación para estimar la biomasa arbórea en los bosques tropicales de Costa Rica. Tecnología en Marcha..

[31] van Best, S., & van Dijk, S. Tropical forests – the facts and figures. Probos Foundation (2020). https://fair-and-precious.org. Downloaded Feb 20, 2021.

[32] FAO *Global Forest Resources Assessment 2020—Key findings*. Rome (2020) 10.4060/ca8753en.

[33] Goodman, R. C. & Herold, M. Why maintaining tropical forests is essential and urgent for a stable climate, in CGD Climate and Forest Paper Series #11 (Center for Global Development Climate and Forest, Washington, DC, 56 pp. (2014). http://www.cgdev.org/publication/why-maintaining-tropical-forests-essential-and-urgent-stable-climate-working-paper-385.

[34] Canet, G. et al. (2015). Avances de los resultados del Inventario Forestal Nacional de Costa Rica. (2015, July 28). Retrieved July 31, 2019 from http://www.sirefor.go.cr/?p=1200.

[35] Cedeño Vindas, M. & Rodriguez Rodriguez, J. L. Medición de Parcelas Permanentes en Bosque Primario Intervenido, Determinacion de Dinámica y Modelos de Crecimiento, Universidad Nacional, Costa Rica, 45 pp (2004).

[36] Holdridge LR (1947). Determination of world plant formations from simple climatic data. Science.

[37] Holdridge, L. R. Ecología basada en zonas de vida. San José, Costa Rica, Editorial IICA; (1967) 206 pp [Translated, Holdrige, L. R., 1967. Life Zone Ecology. Tropical Science Center, San Jose, Costa Rica].

[38] Sánchez-Azofeifa, G.-A. Assessing Land Use/Cover Change in Costa Rica. Ph. D. Dissertation. University of New Hampshire, New Hampshire, 181 pp (1996).

[39] Sánchez-Monge, M. Protocolo de Establecimiento y Medición de Parcelas. Permanentes de Muestreo en Bosque Natural. Red de Parcelas Permanentes de Monitoreo de Ecosistemas Forestales. INISEFOR (2011) (Translated by J. Moy, 2018; Protocol for the Establishment and Measurement of Permanent Sampling Plots in Natural Forest; For the Network of Permanent Plots for the Monitoring of Forest Ecosystems).

[40] Chave J (2005). Tree allometry and improved estimation of carbon stocks and balance in tropical forests. Oecologia.

[41] Chave J (2014). Improved allometric models to estimate the aboveground biomass of tropical trees. Glob. Change Biol..

[42] Cho, P., Mesh, S. & Kay, E. University of Belize Environmental Research Institute. Field Manual for Permanent Sampling Plot Establishment and Re-measurement, 27 pp.

[43] Condit, R. Methods for estimating above-ground biomass of forest and replacement vegetation in the tropics. Center for Tropical Forest Science Research Manual, 73 pp (2008).

[44] Feldpausch TR (2011). Height-diameter allometry of tropical forest trees. Biogeosciences.

[45] Feldpausch TR (2012). Tree height integrated into pantropical forest biomass estimates. Biogeosciences.

[46] Fonseca GW, Federico AG, Rey BJM (2009). Modelos para estimar la biomasa de especies nativas en plantaciones y bosques secundarios en la zona Caribe de Costa Rica. Bosque (Valdivia).

[47] Montero MM, Montagnini F (2005). Modelos alométricos para la estimación de biomasa de diez especies nativas en plantaciones en la región Atlántica de Costa Rica. Recursos Naturales y Ambiente.

[48] Tenorio C, Moya R, Cynthia Salas C, Berrocal A (2016). Evaluation of wood properties from six native species of forest plantations in Costa Rica. Bosque.

[49] Zhou X (2021). Dynamic allometric scaling of tree biomass and size. Nat. Plants.

[50] National Standard. INTE/DN 03: Metodología para la cuantificación y reporte de remociones de gases de efecto invernadero producto de actividades forestales. Methodology for Quantification and Reporting Greenhouse Gas Removals Resulting from Forestry Activities. Instituto de Normas Tecnica de Costa Rica; INTEC, 33 pp (2016).

[51] Eggelston, S., Buendia, L., Miwa, K., Ngara, T. & Tanabe, K., Eds. IPCC Guidelines for National Greenhouse Gas Inventories, Institute for Gobal Environmental Startegies (IGES) for the IPCC (2006). https://www.ipcc.ch/report/2006-ipcc-guidelines-for-national-greenhouse-gas-inventories/.

